# Cycling performance enhancement and injury prevention use an arch support insole with forefoot wedge

**DOI:** 10.1186/1757-1146-7-S1-A102

**Published:** 2014-04-08

**Authors:** Sai-Wei Yang, Po-Hsun Li, Keh-Tao Liu

**Affiliations:** 1 Department of Biomedical Engineering, National Yang-Ming University, Taipei, Taiwan; 2 Global Action Inc- Footdisc®, Taiwan

## 

Bicycle riding is an increasingly popular recreational and competitive activity, however, the more popular the more biking-related injuries. Most of cycling injuries are musculoskeletal related and caused by a combination of inadequate preparation, inappropriate bike fitting, poor technique, and overuse of prolonged uphill biking. However, after well bike fitting, the injury is still existed, the leg alignment and foot types might be an important factor that mainly causes the muscular injury after an appropriate bike fitting. [1,2,3]The purpose of this study was to investigate the efficacy of an arch support insole with/o forefoot wedge in muscle activities and joint loads in order to mimic the musculoskeletal sport injury in cycling and to enhance the performance.

Eleven amateur cyclists were recruited for this study. Vicon motion analysis system, Pedar in-sole foot pressure sensor and Delsys EMG system were used to measure the three-dimensional lower extremity kinematics, kinetics, EMG signal. Each subject was randomly shot four different insoles (Bikepro, off-counter insole with/o arch support and forefoot wedge) with his own bike shoes and bike mounted on a cycle ergometer set to a fixed power of 150W in 75rpm. An One-way ANOVA repeated measurement was used to discriminate the effect of insole material, arch, and forefoot wedge. The results showed that the BikePro significantly increased the ankle varus angle(0.4°, *p*=0.029), the knee internal rotation (1.4°, *p*=0.030), and it significantly decreased the ankle abduction angle (1.2°, *p*=0.047)at the bottom dead center (BDC); reduced the knee sway area by (10.4% , *p*=0.037). Combined with forefoot wedge, it significantly decreased the ankle varus(0.5° , *p*=0.005), and increased the ankle abduction angle (1.2°, *p*=0.005) but without changing the knee trajectory patterns.

**Figure 1:**
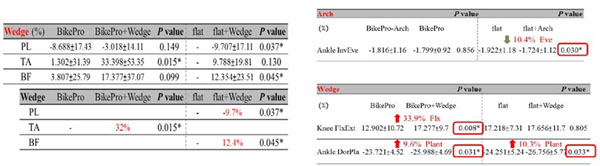
Change of Muscle activities and joint moment in different arch support and wedges

The muscle activation time reduced for the biceps femoris(6.8% , *p*=0.005) in comparision with the off-counter insoles. Combined with wedge it significantly increased the tibialis anterior EMG peak(32% , *p*=0.015) as well as the EMG integral (33%, *p*=0.019), and the integral of biceps femoris was also increased (12.5% , *p*=0.048) when vs. without the wedge. The arch support decreased the peak knee sagittal plane moment on the same performance, increased efficiency during cycling. With the wedge, the high forces found the hallux region and first metatarsal head region which increased the peak knee and ankle moment (Figure 1)

This study suggests the cyclist shall wear proper sports orthotic with arch support and forefoot wedge according to one’s limb alignment, foot type as well as the forefoot angle, in addition to the bike fitting to reduce the overused musculoskeletal related injury.
